# Comparative Transcriptome Analysis in *Taraxacum koksaghyz* to Identify Genes that Determine Root Volume and Root Length

**DOI:** 10.3389/fgene.2021.784883

**Published:** 2022-01-24

**Authors:** Annika Wieghaus, Kai-Uwe Roelfs, Richard M. Twyman, Dirk Prüfer, Christian Schulze Gronover

**Affiliations:** ^1^ Institute of Plant Biology and Biotechnology, University of Münster, Münster, Germany; ^2^ Fraunhofer Institute for Molecular Biology and Applied Ecology (IME), Münster, Germany; ^3^ TRM Ltd., Scarborough, United Kingdom

**Keywords:** *Taraxacum koksaghyz*, root, natural rubber, transcriptomics, breeding

## Abstract

The Russian dandelion (*Taraxacum koksaghyz*, family Asteraceae) produces large amounts of natural rubber in the laticifers of its roots. This species has been proposed as an alternative source of natural rubber to augment or partly replace the rubber tree (*Hevea brasiliensis*) but domestication would require genetic improvement to increase rubber yields and agronomic optimization to facilitate harvesting and processing. Optimization has focused thus far on the size and shape of the roots, the primary storage organ for natural rubber and inulin. However, the corresponding genetic factors are poorly understood. Here we describe the comparative transcriptomic analysis of root tissues from *T. koksaghyz* plant sets featuring different root sizes and shapes, aiming to identify differentially expressed genes correlating with root length or root diameter in the upper root and root tip. The resulting datasets revealed multiple candidate genes for each trait and root part, including a glucan endo-1,3-β-d-glucosidase, an allene oxide synthase 3, and a TIFY10A/JAZ1 homolog. These three genes were tested by qRT-PCR in outdoor-grown plants with diverse root morphology, and the expression of two genes correlated with the appropriate root morphotype, confirming the effectiveness of our method. We evaluated the candidate genes to gain insight into their potential functions in root development. Such candidate genes could be suitable for marker-assisted breeding programs in the future.

## Introduction

The development of new crop varieties and the improvement of existing ones requires the identification and subsequent introduction of new traits such as pest and disease resistance, tolerance of extreme climatic conditions, and higher yields. Although research has focused on food and feed crops to ensure food security, the same objectives mentioned previously apply to crops that serve as feedstock for industrially relevant raw materials. One example is the Russian dandelion (*Taraxacum koksaghyz*), a member of the family Asteraceae originally native to Kazakhstan, which produces large amounts of natural rubber and inulin in its roots and has therefore been proposed as an alternative source of both materials (Krotkov, 1945; Whaley and Bown, 1947; van Beilen and Poirier, 2007a, 2007b).

The Russian dandelion was first cultivated as a crop during World War II when imports of natural rubber produced from the primary source (the rubber tree *Hevea brasiliensis*) were interrupted. But these attempts were abandoned in the early 1950s when the reinstatement of rubber imports made the alternative source economically less attractive (van Beilen and Poirier, 2007b; Schulze Gronover et al., 2011). More recently, interest in alternative sources of natural rubber has returned due to increasing demand and the disadvantages of the rubber tree (Cornish, 2017). *H. brasiliensis* is a slow-growing species with a long breeding cycle, is restricted to specific climate regions, and mainly grows in monoculture plantations with narrow genetic diversity and a high susceptibility to pathogens (Lieberei, 2007). The Russian dandelion produces natural rubber of comparable quality to the rubber tree, with yields of up to 15% of the root dry weight (van Beilen and Poirier, 2007a). Furthermore, up to 50% of the root dry weight is the storage carbohydrate inulin (Uhlmann, 1951). This is used as a sweetener and additive in the food industry and serves as feedstock for bioethanol production (Ohta et al., 1993; Roberfroid, 2005). The current main industrial source of inulin is chicory (Van Den Ende et al., 2004).

The Russian dandelion is a fast-developing annual plant that can produce large amounts of root biomass when cultivated in temperate climate zones (van Beilen and Poirier, 2007b). If large-scale regional cultivation were established, this species could therefore meet a large proportion of the current demand for natural rubber, reducing the vulnerability of supply chains and the carbon footprint of rubber imports from South and Southeast Asia. Several studies have already assessed the performance of *T. koksaghyz* plants in terms of biomass accumulation, rubber and inulin content, and cultivation time and conditions (Arias et al., 2016a, 2016b; Kreuzberger et al., 2016; Eggert et al., 2018). However, the agronomic performance requires further optimization to increase yields and facilitate harvesting and processing in order to make the crop more competitive. One way to achieve these aims is to optimize the root volume (increasing the capacity for rubber and inulin accumulation) and root shape (medium length with more volume in the upper part, to facilitate harvesting and extraction).

Root growth is influenced by internal and external factors, particularly the interactions between phytohormones such as brassinosteroids (Roddick et al., 1993; Clouse et al., 1996), jasmonate (Dathe et al., 1981; Staswick et al., 1992) and auxins (Chadwick and Burg, 1967), as well as various signaling peptides (Yamada and Hsiao, 2021). In *T. koksaghyz*, only the influence of the small signaling peptide TkRALF1 has been investigated thus far (Wieghaus et al., 2019). Little is known about the hormonal response to environmental conditions such as phosphorous and nitrogen availability in the rhizosphere, the root microbiome, or abiotic stresses such as water deprivation. The same applies to the corresponding physiological and morphological adaptations in the roots, thus limiting the value of population-wide analysis of root growth to identify correlations between single genes and phenotypes.

To address this knowledge gap, we analyzed the transcriptome of Russian dandelion plant sets differing in terms of root length and root volume. Massive analysis of cDNA ends (MACE) was chosen for transcriptome generation because this technique is able to detect rare transcripts at significantly lower sequencing depth than classical RNAseq and is not susceptible to bias that originates from different transcript lengths (Zawada et al., 2014; Bojahr et al., 2016).

We used a forward genetics approach to identify genes associated with beneficial root phenotypes suitable for future breeding programs. Here we describe the first candidate genes, and more importantly provide sets of candidate genes related specifically to *T. koksaghyz* root volume or root length.

## Methods

### Plant Cultivation and Processing

The plants used in this study were provided by Dr. Fred Eickmeyer, ESKUSA GmbH, and originate from a wild-type population of highly heterozygous individuals which is due to the fact that *T. koksaghyz* is a facultative outcrossing, sexually reproducing species. Seeds were collected from a large set of *T. koksaghyz* plants grown under the greenhouse conditions described below after open pollination. Seeds were sown in rows in large buckets containing a mixture of 70% topsoil (Botanical Garden Münster, Germany) and 30% sand. Seeds started to germinate after 3 days and only seedlings that had emerged on days three or four were used further. The plants were grown in the greenhouse at 22–25°C (day) and 14–18°C (night) with a 16-h photoperiod (20 klx light intensity). A first set of plants was grown for transcriptomic analysis. We harvested the plants after 12 weeks and assigned them to three pools of 21–36 plants based on root morphology: thick roots of medium length (pool A), short and thin roots (pool B), or long and thin roots (pool C) (detailed classification in Supplementary Table S1). The roots were measured lengthwise using a ruler and the diameter was determined using digital calipers. Each root was then divided into two parts: proximal (upper root including lateral roots) and distal (root tip up to the first emerging lateral root). The parts were separately frozen in liquid nitrogen, lyophilized and ground to powder for transcriptome analysis.

We then cultivated a second set of wild-type *T. koksaghyz* plants as described above, but the pots were placed outside so that the plants were exposed to natural weather and day length. This second set of plants was used to validate first possible candidate genes gained from the transcriptomic analysis of the first set of wild-type plants. Quantitative real-time PCR (qRT-PCR) was used for the gene expression analysis of individual plants harvested from this second set. The roots were harvested after 12 weeks and measured as described above. We then processed whole roots without division into proximal and distal segments, using the same process of freezing, lyophilization and grinding as described above. The whole-root material was used to provide sufficient amounts of RNA for purification and cDNA synthesis followed by qRT-PCR.

For spatial gene expression analysis, *T. koksaghyz* plants were cultivated for 12 weeks as described above in the greenhouse. The plants were harvested individually and subsequently separated into flower, peduncle, leaf, root, and latex. The tissue material was then processed as described above.

### MACE

Massive analysis of cDNA ends (MACE) libraries (one library per pool) were prepared and sequenced by GenXPro (Frankfurt, Germany) as previously described (Zawada et al., 2014; Bojahr et al., 2016). The sequencing data generated by MACE are deposited in the SRA under the bioproject accession PRJNA783530.

### Determination of the Natural Rubber Content

The natural rubber content of the root material used to prepare MACE libraries was quantified by accelerated solvent extraction as previously described (Benninghaus et al., 2020). The natural rubber content was determined gravimetrically by weighing the empty collection vials and those containing dried hexane extracts, then calculating the weight percentage as a proportion of root dry weight using the exact quantity of root material.

### Quality Control, Read Mapping and Annotation

To increase mapping accuracy, low-quality bases (Phred score <20) were trimmed from the 3′ end of all reads using *cutadapt* v1.16 (Martin, 2011). Given the nature of the MACE technique, reads originate from 50 to 800 bp upstream of the 3′ ends of the transcripts, which often results in reads containing poly(A) tail sequences. Therefore, following the previous step, a *de novo* script was used to detect and remove poly(A) tail sequences, which would otherwise interfere with mapping. Finally, reads <25 bp were discarded using *cutadapt* to prevent nonspecific mapping. Quality-trimmed reads were mapped to the reference genome (Lin et al., 2017) using *segemehl* (Hoffmann et al., 2009) with split reads included.

### Differential Expression Analysis

Raw read counts were generated using *bamutils* v0.5.9 (Breese and Liu, 2013) with partially counted multiple hits, which represented the basis for differential expression analysis using the MA plot method with random sampling (MARS) in the *DEGSeq* R package v1.32.0 (Wang et al., 2009). Transcripts were annotated by using the NCBI BLAST suite v2.7.1 (Altschul et al., 1990) to screen the NCBI non-redundant proteins (O’Leary et al., 2016) and UniProtKB/SWISS-PROT (Bateman et al., 2017) databases. Transcripts with a normalized one-fold or greater differential expression (log-2-fold change ≥ 1 or ≤ −1, respectively) combined with a *p*-value less than 0.001 (*p* < 0.001) were considered significant and the corresponding genes were defined as differentially expressed genes (DEGs). Candidate genes for root length and diameter were selected according to following conditions: transcripts that were differentially expressed in pools A vs B and B vs C but not A vs C were considered as candidates for root length, whereas those differentially expressed in pools A vs B and A vs C but not B vs C were considered as candidates for root diameter.

### Data Analysis

Multi-dimensional scaling (MDS) plot analysis was performed using limma R package v3.46.0 (Ritchie et al., 2015). Venn diagrams were prepared using Vennplex (https://www.nia.nih.gov/research/labs/vennplex; Cai et al., 2013). Heatmaps showing differential gene expression were generated using heatmapper (http://www.heatmapper.ca; Babicki et al., 2016). Gene Ontology (GO) enrichment was analyzed using WEGO (http://wego.genomics.org.cn; Ye et al., 2018, 2006) with all transcripts in the corresponding root part used as the background. Glycosylphosphatidylinositol (GPI) anchors, protein domains and signal peptides were predicted using PredGPI (Pierleoni et al., 2008), CD search (Lu et al., 2020) and SignalP v5.0 (Almagro Armenteros et al., 2019), respectively. Phylogenetic trees were constructed using MEGA X (Kumar et al., 2018) and graphically rendered using iTOL v6.3.1 (Letunic and Bork, 2021).

### Extraction of Total RNA and cDNA Synthesis

Total RNA was extracted from *T. koksaghyz* root material using the innuPREP RNA Mini Kit (Analytik Jena, Jena, Germany) according to the manufacturer’s instructions, and cDNA was synthesized using PrimeScript RT Master Mix (TAKARA, Clontech, Sain-Germain-en-Laye, France) according to the manufacturer’s instructions.

### Gene Expression Analysis by qRT-PCR

Quantitative real-time PCR was carried out as previously described (Laibach et al., 2015) with slight modifications. The elongation factor 1α (*TkEF1α*) and ribosomal protein L27 (*TkRP*) reference genes were used for the normalization of gene expression as described before (Niephaus et al., 2019; Benninghaus et al., 2020). The qRT-PCR data were analyzed, and normalized expression levels were calculated using Bio-Rad CFX Manager v3.1 (Bio-Rad Laboratories, Hercules, CA, United States). All oligonucleotides used for qRT-PCR analysis are listed in Supplementary Table S2 along with corresponding primer efficiencies. For determination of primer efficiencies, a standard curve using a serial dilution of a representative cDNA sample was generated and subsequently analyzed using Bio-Rad CFX Manager v3.1. Efficiencies are calculated by the software as described by Pfaffl (2001) and Vandesompele et al. (2002), converted to percentage efficiency.

### Statistical Analysis

Data analysis using the two-tailed *t*-test, Kolmogorov-Smirnov normal distribution analysis, Grubbs’s test for the identification of outliers, and correlation studies (linear fit with calculation of Pearson’s correlation coefficient) was carried out using OriginPro 2019b (OriginLab Corporation, Northampton, MA, United States).

## Results

### Transcriptome Data Generation and Processing

**FIGURE 1:**
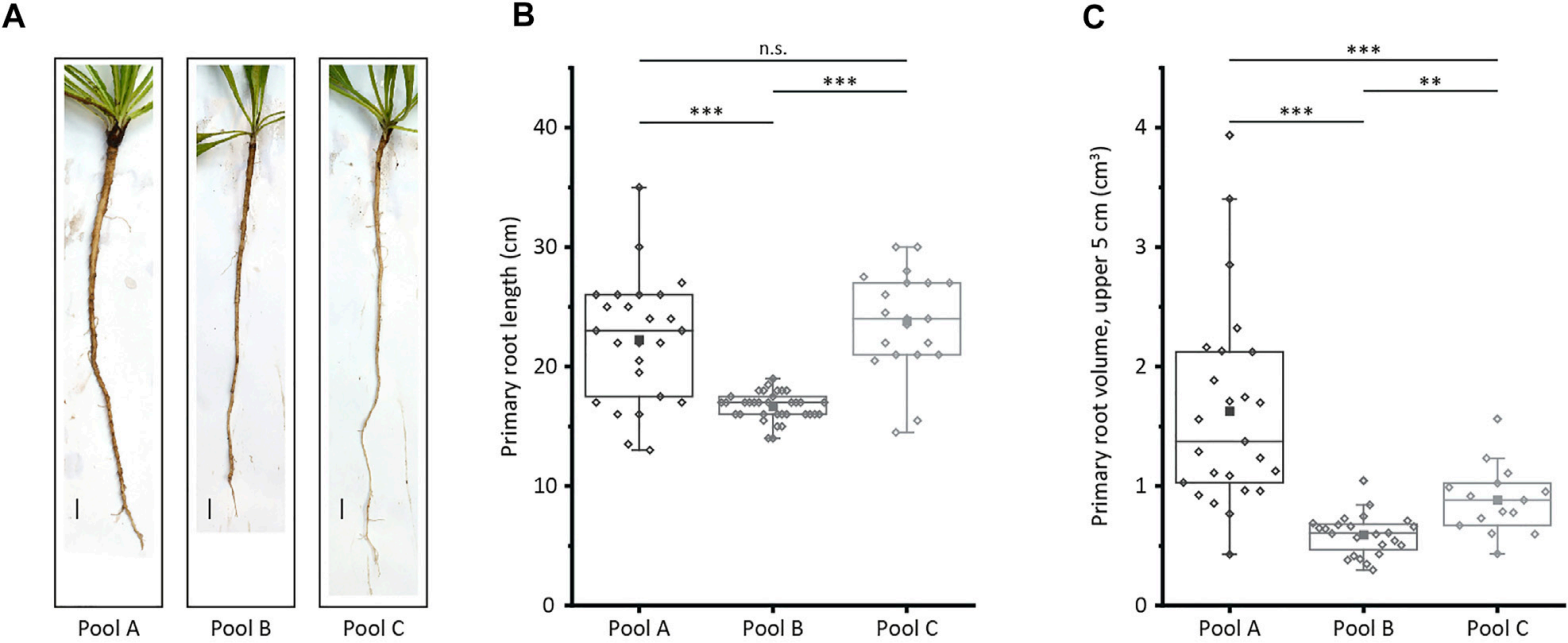
Characterization of the three plant pools selected for MACE. The primary root length and the volume of the upper 5 cm of the root in 12-week-old *T. koksaghyz* wild-type plants were determined after harvesting. The plants were then assigned to three plant pools **(A)** Pictures of plants representing the three pools. Scale bar = 1 cm **(B)** Primary root length **(C)** Volume of the upper 5 cm of the primary root, determined by measuring the root diameter immediately below the leaf rosette and 5 cm further down. The boxes delimit values from the 25th to the 75th percentile of each dataset, the horizontal line in the box represents the median, the filled square represents the mean, and the lower and upper whiskers represent values that differ least from 25th percentile –1.5*IQR or 75th percentile +1.5*IQR, respectively (n = 15–35 plants, normal distribution confirmed using the Kolmogorov-Smirnov test, statistical significance confirmed using a two-tailed *t*-test; n. s = not significant; *p < 0.05; **p < 0.01; ***p < 0.001).

To identify genes that potentially affect *T. koksaghyz* root morphology, we generated a set of wild-type plants and subdivided it into three pools of plants with different root characteristics. The root morphology of the pools differed significantly in terms of primary root length, diameter directly below the leaf rosette, and the volume of the upper 5 cm, which was calculated by determining the root volume at two defined points (Figure 1, Supplementary Table S1). However, all three pools produced comparable amounts of natural rubber (Supplementary Table S1). Pool A comprised plants with medium to long roots that were also thick (large volume), representing the most desirable root phenotype for biomass accumulation as well as harvesting and handling. Pool B comprised plants with short and thin roots, whereas pool C comprised plants with long and thin roots.

We compared gene expression profiles between pools in two segments of the root. Each complete root was divided into the proximal segment (upper root including lateral roots) and distal segment (root tip up to the first emerging lateral root), resulting in a total of six pooled samples. *T. koksaghyz* is a sexually reproducing and self-incompatible species, so wild-type plants show a high degree of genetic diversity. By pooling plants with similar root morphology prior to transcriptome analysis, we aimed to reduce the background noise and emphasize transcripts that are differentially expressed between pools, thus capturing those representing genes related to the development of specific root characteristics.

We prepared and sequenced cDNA libraries from all six samples using MACE technology (Kahl et al., 2012). The six libraries contained between 4.65 × 10^6^ and 6.68 × 10^6^ single-end raw reads of 16–145 bp in length, which were further processed using a custom Python script to remove poly(A) tails. Following the removal of adapters and the trimming of low-quality bases, the libraries contained between 4.55 × 10^6^ and 6.48 × 10^6^ clean reads, 86.3–87.3% of which mapped to the reference genome (Lin et al., 2017), and 74.3–78.5% of which were uniquely matched (Table 1). We obtained 30,955 unique transcripts from the six libraries, which were then annotated using the NCBI and UniProt databases. To confirm sufficient reduction of background noise by pooling, we constructed an MDS plot, which showed the clear separation of all samples (Supplementary Figure S1).

**TABLE 1:** Summary of MACE alignment statistics in six libraries mapped to the reference genome.

	Pool A		Pool B		Pool C	
	Upper root	Root tip	Upper root	Root tip	Upper root	Root tip
Total base pairs	535,992,378	444,400,123	600,607,725	754,363,862	508,730,146	777,887,360
Total clean reads	5,288,641	4,551,524	4,941,227	6,265,938	4,807,513	6,479,601
Mapped reads	4,619,116 (87.34%)	3,956,571 (86.93%)	4,293,960 (86.90%)	5,415,759 (86.43%)	4,179,649 (86.94%)	5,593,472 (86.32%)
Unique matches among mapped reads	3,608,474 (78.12%)	2,941,045 (74.33%)	3,370,356 (78.49%)	4,221,179 (77.94%)	3,202,090 (76.61%)	4,302,574 (76.92%)
Multi-position among mapped reads	750,024 (16.24%)	798,720 (20.19%)	654,546 (15.24%)	856,771 (15.82%)	745,256 (17.83%)	928,985 (16.61%)
Split-reads among mapped reads	260,618 (5.64%)	216,806 (5.48%)	269,058 (6.27%)	337,809 (6.24%)	232,303 (5.56%)	361,913 (6.47%)
Unmapped reads	669,525 (12.66%)	594,953 (13.07%)	647,267 (13.10%)	850,179 (13.57%)	627,864 (13.06%)	886,129 (13.68%)

### Identification Root Volume and Root Length Candidate Genes by Differential Gene Expression Analysis

**FIGURE 2:**
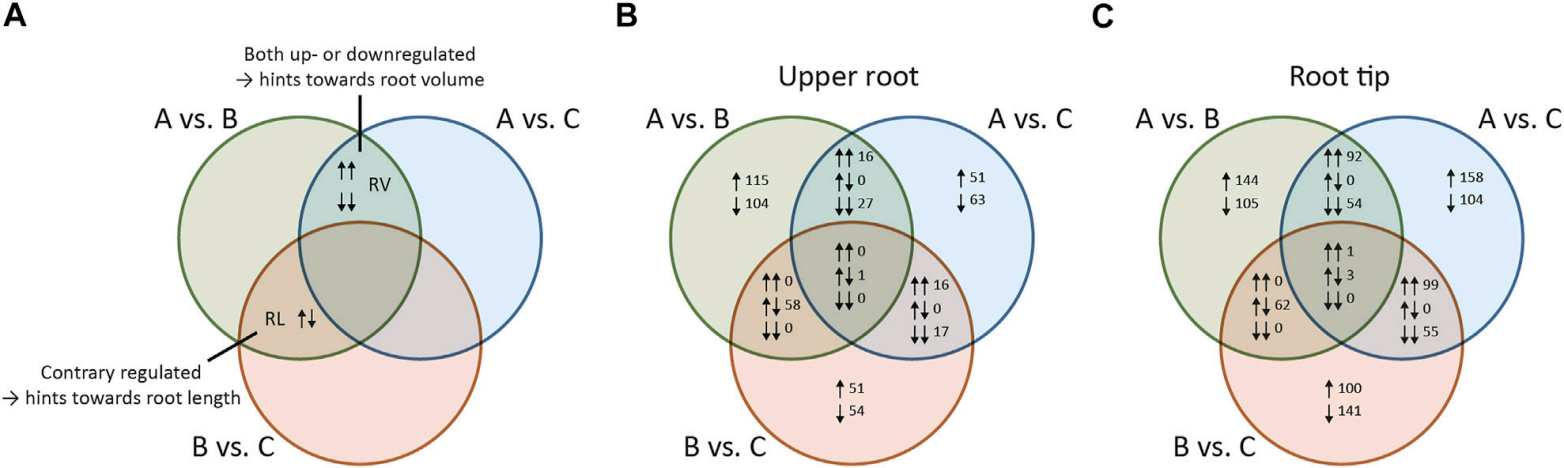
Venn diagram of differentially expressed genes (DEGs) identified by pairwise comparison of plant pools differing in root morphology. All pairwise comparisons between the pools were set in relation to each other based on log2 fold change values in order to identify shared DEGs **(A)** Schematic Venn diagram illustrating the identification of DEGs related to primary root volume or primary root length **(B)** Venn diagram showing DEGs identified by comparison among the pools in the upper root **(C)** Venn diagram showing DEGs identified by comparison among the pools in the root tip. Pool A = plants with medium to long and thick roots (high value for primary root length and volume in the upper 5 cm of the primary root). Pool B = plants with short, thin roots. Pool C = Plants with long, thin roots. RV = DEGs influencing primary root volume; R = DEGs influencing primary root length; ↑ = DEGs with positive log2 fold changes (more abundant in the first pool in each comparison); ↓ = DEGs with negative log2 fold changes (more abundant in the second pool in each comparison); ↑↑ = DEGs with positive log2 fold changes for all comparisons (more abundant in the first pool of each comparison); ↓↓ = DEGs with negative log2 fold changes for all comparisons (more abundant in the second pool of each comparison); ↑↓ = DEGs with opposing log2 fold change values (positive in one comparison but negative in the other).

Next, we identified the genes with significant differential expression between pools, defined as those with a log2 fold change (l2fc) ≥ 1 or ≤ –1 (*p* < 0.001) in any pairwise comparison. We then constructed a Venn diagram and used this to find genes related to either root volume or root length (Figure 2A).

Genes with significant differential expression when comparing pool A vs B and pool A vs C were considered as candidates influencing root volume (RV in Figure 2A) because the volume of the roots in pool A was significantly higher compared to both pool B and pool C. The initial list of candidates was reduced by excluding those also showing differential expression when comparing pool B vs C because the roots in these pools were similar in volume. By applying this last criterion, we arrived at a final set of RV candidate genes (schematic description in Supplementary Figure S2). Genes with significant differential expression when comparing pool A vs B and pool B vs C were considered as candidates influencing root length (RL in Figure 2A) because the roots in pools A and C were significantly longer than the roots in pool B. As above, we narrowed the initial list of candidates by excluding those also showing differential expression between pools A and C because the roots in these pools were similar in length. By applying this last criterion, we arrived at a final set of RL candidate genes (schematic description in Supplementary Figure S2). The Venn diagrams showed that DEGs defining the RV intersection were either upregulated or downregulated in both comparisons (A vs B *and* A vs C), but no counter-regulated genes were observed. In contrast, all DEGs in the RL intersection were counter-regulated (either upregulated in A vs B but downregulated in B vs C or vice versa).

**FIGURE 3:**
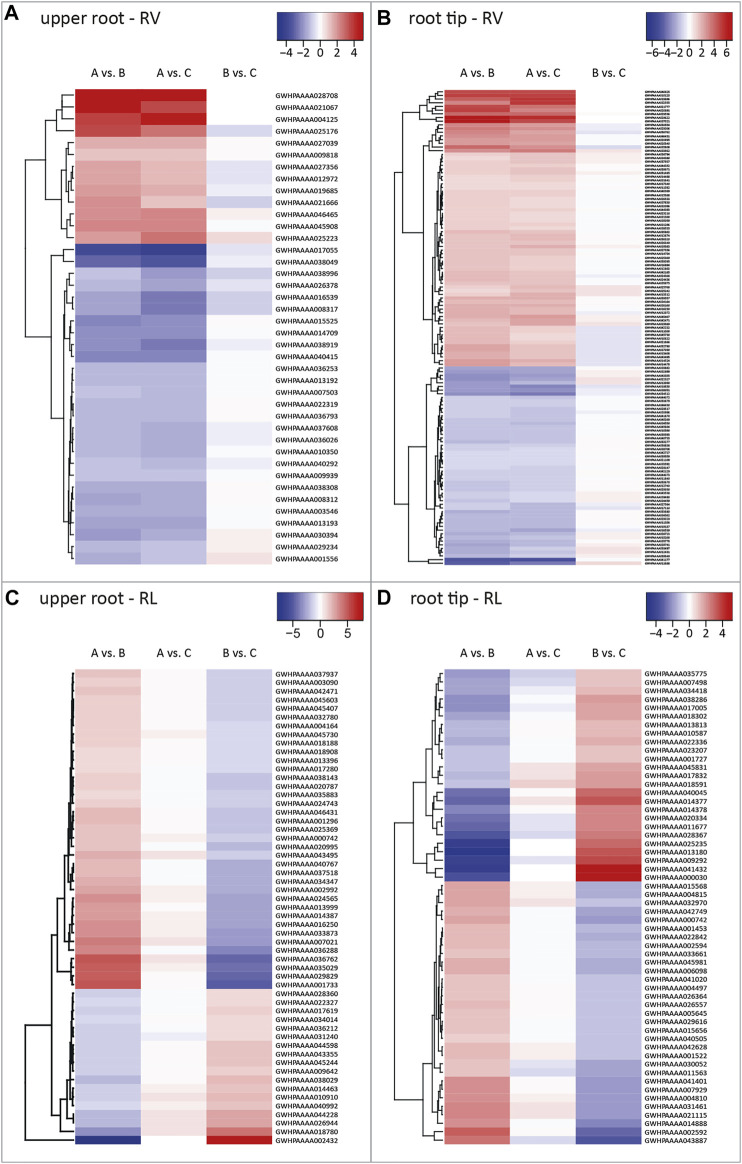
Heat maps of root volume (RV) and root length (RL) candidate genes. Heat maps visualizing all RV and RL candidate genes identified in different root parts with their corresponding log2 fold change values for pairwise comparisons among the three plant pools. Each row depicts the log2 fold change values of the comparisons with blue, white and red boxes representing negative, close-to-zero and positive values, as indicated by the corresponding scale bars. Shown are RV candidate genes identified in **(A)** the upper root and **(B)** the root tip, as well as RL candidate genes identified in **(C)** the upper root, and **(D)** the root tip. Each row shows log2 fold change values of pairwise comparisons between the plant pools. Pool A = plants with medium to long and thick roots (high value for primary root length and volume in the upper 5 cm of the primary root). Pool B = plants with short, thin roots. Pool C = Plants with long, thin roots. RV = DEGs influencing primary root volume. RL = DEGs influencing primary root length.

We identified 573 DEGs for the upper root segment. We extracted 40 RV candidate genes from the 43 DEGs in the RV intersection by manually excluding genes showing differential expression when comparing pool B vs C with *p* > 0.001, because we considered these l2fc values as not statistically robust (Figure 2B). The corresponding heat map revealed that 33% of the RV candidate genes were expressed more strongly in roots with a higher volume (pool A) compared to those with a lower volume (pools B and C) as indicated by the positive l2fc values (Figure 3A). We also extracted 55 RL candidate genes from the 58 DEGs in the RL intersection (Figure 2B), 67% of which were expressed at higher levels in long roots (pools A and C) compared to short roots (pool B) (Figure 3C).

We identified 1,118 DEGs for the root tip, including 129 RV candidate genes from 146 DEGs assigned to the RV intersection and 56 RL candidate genes from 62 DEGs assigned to the RL intersection (Figure 2C). We found that 58% of the RV candidate genes were expressed at higher levels in roots with a larger volume (Figure 3B), and 55% of the RL candidate genes were expressed at higher levels in longer roots (Figure 3D). All DEGs and candidate genes are listed in Supplementary Table S3.

### GO Term Enrichment of the RV and RL Candidate Genes

GO term enrichment analysis was carried out to determine the putative functions of the RV and RL candidate genes. Overall, we observed a similar distribution of second-level GO terms for all sets of candidate genes in the three main GO categories: cellular component, molecular function and biological process (Supplementary Figure S3). The most abundant GO terms assigned in the cellular component category were *cell*, *cell part*, *organelle*, *organelle part*, and *membrane*. For the molecular function category, the most represented GO terms were *catalytic activity* and *binding*. For the biological process category, the most represented GO terms were *cellular process*, *metabolic process*, *response to stimulus*, *biological regulation*, and *regulation of biological process*. Several GO terms were significantly enriched or underrepresented at level 2 (Supplementary Figure S3). Among the RV candidate genes in the root tip dataset, the cellular component *extracellular region* was enriched compared to all transcripts, whereas multiple terms (*cell*, *cell part*, *membrane*, and *membrane part* in the cellular component category; *cellular process*, *metabolic process*, *biological regulation*, and *cellular component organization or biogenesis* in the biological process category) were underrepresented compared to all transcripts. In addition to this general characterization, more specific GO terms at deeper levels were also evaluated by enrichment analysis (Table 2). Compared to all transcripts, *hydrolase activity* was enriched in the RV candidate genes of the upper root. Several GO terms describing response to different stimuli (chemical, hormone, oxygen-containing compound) were enriched in the RV candidate gene set in the root volume. Among others, *intrinsic component of membrane*, *integral component of membrane*, *regulation of metabolic process*, *cellular metabolic process*, and *cellular macromolecule metabolic process* were underrepresented in this candidate gene set. For the GO term distribution in the RL candidate genes of the upper root we found components not associated with membrane-bound organelles to be enriched (*non-membrane-bounded organelle*, *intracellular non-membrane-bounded organelle*) and vice versa (*membrane-bounded organelle*, *intracellular membrane-bounded organelle*). *Cell periphery* was enriched in the RL candidate genes of the root tip.

**TABLE 2:** Summary of the GO enrichment analysis at deeper GO levels.

Candidate gene set	GO term (↑/↓)	GO class and level
upper root - RV candidate genes	hydrolase activity (**↑**)	molecular function, level 3
root tip - RV candidate genes	intrinsic component of membrane (**↓**)	cellular component, level 3/4
	integral component of membrane (**↓**)	cellular component, level 4
	nuclear part (**↓**)	cellular component, level 4
	response to endogenous stimulus (**↑**)	biological process, level 3
	cellular component organization (**↓**)	biological process, level 3
	regulation of metabolic process (**↓**)	biological process, level 3/4
	response to chemical (**↑**)	biological process, level 3
	cellular metabolic process (**↓**)	biological process, level 3
	primary metabolic process (**↓**)	biological process, level 3
	response to acid chemical (**↑**)	biological process, level 4
	response to hormone (**↑**)	biological process, level 4
	drug metabolic process (**↑**)	biological process, level 4
	cellular macromolecule metabolic process (**↓**)	biological process, level 4
	response to oxygen-containing compound (**↑**)	biological process, level 4
upper root - RL candidate genes	non-membrane-bounded organelle (**↑**)	cellular component, level 3
	membrane-bounded organelle (**↓**)	cellular component, level 3
	intracellular non-membrane-bounded organelle (**↑**)	cellular component, level 4
	intracellular membrane-bounded organelle (**↓**)	cellular component, level 4
	small molecule metabolic process (**↑**)	biological process, level 3
root tip - RL candidate genes	cell periphery (**↑**)	cellular component, level 3/4

↑, enriched in candidate genes compared to all transcripts; ↓, underrepresented in candidate genes compared to all transcripts.

### Transcriptome Characterization

To characterize our transcriptome dataset further, we searched for typical factors affecting root growth and development like transcription factors, hormone pathway genes and cell wall/glucan biosynthesis/degradation genes based on the UniProt annotation (Supplementary Table S4–S6).

We identified 10 differentially expressed transcription factors which mostly originate from the volume and length trait groups in the root tip. Members of this set include two basic helix-loop-helix transcription factors (Transcription factor bHLH62, GWHPAAAA007503; Transcription factor bHLH145, GWHPAAAA042185), a transcription factor of the TCP family (Transcription factor TCP8, GWHPAAAA044352), an AP2 domain containing or RAP2 (related to AP2) family protein (Ethylene-responsive transcription factor RAP2-4, GWHPAAAA002086) and Protein LIGHT-DEPENDENT SHORT HYPOCOTYLS 10 (GWHPAAAA017340) of the ALOG family. Other members of the ALOG family were proposed to be developmental regulators because they promote cell growth in *Arabidopsis thaliana* (Zhao et al., 2004; Cho and Zambryski, 2011). This might indicate that the member we identified in our dataset plays a role during root development in *T. koksagyhz*.

We also found 16 genes associated with or induced by certain hormone pathways, including brassinosteroids (Protein EXORDIUM, GWHPAAAA001556 and GWHPAAAA007090; Shaggy-related protein kinase eta, GWHPAAAA008535), auxin (Auxin response factor 2A, GWHPAAAA014478; Remorin, GWHPAAAA014754) and jasmonates (Protein TIFY 10A, GWHPAAAA022842; Protein SGT1 homolog B, GWHPAAAA001300; Allene oxide synthase 3, GWHPAAAA036026). Most genes were found in the volume trait groups of the upper root and root tip. Protein EXORDIUM was shown to promote growth in the root and shoot when overexpressed in *A. thaliana* (Coll-Garcia et al., 2004) and Auxin response factor 2A downregulation increased root branching in tomato (Hao et al., 2015).

The last group comprised 11 genes encoding cell wall/glucan biosynthesis/degradation factors. Members of this group included not only glucan endo-1,3-β-D-glucosidases (β-1,3-glucanases; GWHPAAAA003778, GWHPAAAA042727, GWHPAAAA038049, GWHPAAAA027039) and xyloglucan endotransglucosylases (GWHPAAAA046431, GWHPAAAA046431, GWHPAAAA013192, GWHPAAAA013193), but also a cellulose synthase A catalytic subunit 6 (UDP-forming)-like gene (GWHPAAAA015656). The latter was previously found to play a crucial role in root and hypocotyl elongation in *A. thaliana* (Hauser et al., 1995; Fagard et al., 2000) and is also associated with the root length trait of the root tip in our dataset.

### Verification of Candidate Genes by qRT-PCR

To confirm the suitability of our approach for root transcriptome analysis, we generated an independent set of wild-type *T. koksaghyz* plants (Supplementary Table S7). These plants were grown outside under natural climate conditions to approximate field growth. The roots were harvested individually after 12 weeks. Subsequently, qRT-PCR analysis was performed using primers specific for a small set of genes from the previous step, which were not obviously associated with root growth, as our goal was to identify novel factors influencing root morphology. They were also selected to represent various types of developmental and cellular mechanisms.

**FIGURE 4:**
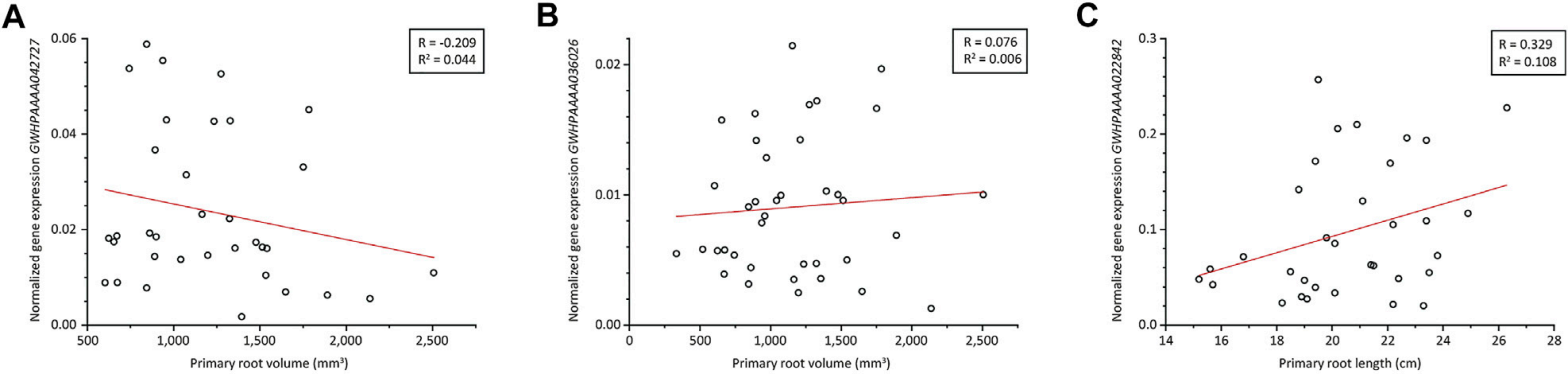
Expression analysis of three candidate genes identified in the *T. koksaghyz* root transcriptome. Gene expression in single *T. koksaghyz* wild-type plants was determined by qRT-PCR. Normalized gene expression of *GWHPAAAA042727* **(A)** and *GWHPAAAA036026* **(B)** was plotted against the primary root volume in the upper 5 cm, and normalized gene expression of *GWHPAAAA022842* **(C)** was plotted against the primary root length. Normal distribution of all parameters was confirmed using the Kolmogorov-Smirnoff-test, and outliers were identified using Grubbs’s test. Red line = regression line; R = Pearson’s R correlation coefficient; *R* ^2^ = coefficient of determination; n = 33–38.

*GWHPAAAA042727* was differentially expressed in the root tip and was annotated as a glucan endo-1,3-β-d-glucosidase (β-1,3-glucanase; UniProt ID Q94G86). This gene was strongly expressed in thin roots (pools B and C) but only weakly expressed in thick roots (pool A) and therefore represented a putative negative regulator of root volume. This was confirmed by qRT-PCR (Figure 4A). Additionally, spatial gene expression analysis showed the highest expression in roots (Supplementary Figure S4A). Protein domain analysis revealed a glycoside hydrolase family 17 (GH-17) domain spanning amino acids 23–343 and an X8/CBM43 superfamily domain spanning amino acids 372–456. The first part of the N-terminus corresponded to a signal peptide with a cleavage site between residues 21 and 22, but no GPI anchor site was predicted, suggesting the protein is not attached to the membrane but probably binds to carbohydrates through its X8/CBM43 domain (Barral et al., 2005). Phylogenetic analysis (Supplementary Figure S5) revealed that this β-1,3-glucanase belongs to the α-clade characterized by a diverse set of protein domain structures and expression patterns (Doxey et al., 2007). Like approximately one third of the β-1,3-glucanases in this clade, the *T. koksaghyz* protein represents protein domain architecture type II, consisting of a signal peptide, a GH-17 domain and an X8/CBM43 domain.

*GWHPAAAA036026* was differentially expressed in the proximal root and was annotated as allene oxide synthase 3 (TkAOS3; UniProt ID Q8GZP5). This gene was also expressed at higher levels in pools B and C compared to pool A and thus represented another putative negative regulator of root volume. Amino acid sequence analysis showed that it shares the same characteristics as other CYP74 family P450 cytochromes, lacking the threonine residue required for the binding and activation of oxygen in the I-helix of P450 monooxygenases (Chapple, 1998). Instead, the small hydrophobic residue valine is found at this position, which is common for CYP74 proteins (Itoh et al., 2002). TkAOS3 also fulfils the CYP74 consensus sequence for cysteinyl heme ligand residues near the C-terminus with the sequence NKQC (A/**P**)(G/**A**)K(**D**/N)XV (Itoh et al., 2002). An unrooted maximum-likelihood tree (Supplementary Figure S6) containing several members of the CYP74 family from different species was constructed to assign TkAOS3 to one of the four CYP74 subfamilies. TkAOS3 clustered with other members of CYP74 subfamily C, which show high affinity for 9-hydroperoxides of linolenic or linoleic acid (9-HPOT/9-HPOD) derived from 9-lipoxygenase (9-LOX) activity, yielding the corresponding allene oxides (9,10-EOD/9,10-EOT) (Itoh et al., 2002; Stumpe et al., 2006). These unstable intermediates are likely to undergo spontaneous cyclization (forming 10-OPDA/10-OPEA) or hydrolysis (forming α-ketol/γ-ketol). However, the tomato AOS3 gene (*LeAOS3*) is thought to encode a multifunctional enzyme that can also catalyze the cyclization or hydrolysis of the intermediate to form *rac-cis*-10-OPEA or *9R*-α-ketol, respectively (Grechkin et al., 2008). The expression of AOS3 is restricted to the roots in tomato (Itoh et al., 2002) and to the sprouting eyes, stolons, tubers and roots in potato (Stumpe et al., 2006). Our qRT-PCR analysis did not find any correlation because the expression of *GWHPAAAA036026* did not negatively correlate with root volume (Figure 4B) as initially suggested by the MACE dataset. Among the different plant tissues, we observed the highest gene expression in latex, which is highly abundant in root tissue. In all other examined tissues, *GWHPAAAA036026* is only weakly expressed in comparison to latex (Supplementary Figure S4B).

*GWHPAAAA022842* was differentially expressed in the root tip and was annotated as a homolog of TIFY10A/JAZ1 (UniProt ID Q9LMA8). This gene was expressed at significantly higher levels in pools A and C compared to pool B, and was thus considered a candidate for a positive regulator of root length. Our qRT-PCR analysis in single plants revealed a positive correlation with the primary root length (Figure 4C), consistent with the MACE dataset. *GWHPAAAA022842* is expressed in almost all tissues except flowers at a moderate level, and the highest expression was observed in the peduncle (Supplementary Figure S4C). The protein contains a TIFY domain (spanning residues 106–138) with a TIFLNG motif differing slightly from the consensus sequence [TIF (F/Y)XG] (Vanholme et al., 2007). It also features a Jas/CCT domain (spanning residues 182–207). A maximum likelihood tree was constructed from *A. thaliana* TIFY/JAZ proteins, revealing that our protein is closely related to AtTIFY10A and AtTIFY10B (Supplementary Figure S7). AtTIFY10A/JAZ1 belongs to the JAZ family of repressors that target transcription factors such as MYC2, MYC3 and MYC4 (Chini et al., 2007; Fernández-Calvo et al., 2011). These transcription factors promote the expression of several early jasmonate response genes and are involved in defense responses as well as developmental processes such as lateral root formation (Yadav et al., 2005; Grunewald et al., 2009; Kazan and Manners, 2013). The degradation of JAZ proteins depends on jasmonyl-isoleucine (JA-Ile)-mediated interactions with COI1 through its Jas domain, which is part of the SCF(COI1) E3 ubiquitin ligase complex, and subsequent processing by the 26S proteasome (Thines et al., 2007). The TIFY domain of JAZ1 interacts with NINJA/AFPH2, which recruits TOPLESS to repress MYC2 (Pauwels et al., 2010). In turn, the expression of multiple JAZ genes (including *JAZ1*) is promoted by MYC2 as well as the closely related transcription factors MYC3 and MYC4, resulting in a negative feedback loop to attenuate the jasmonate signal (Chini et al., 2007; Niu et al., 2011).

## Discussion

Domestication of the Russian dandelion (*T. koksagyhz*) would provide a sustainable source of natural rubber. However, it is first necessary to develop breeding programs to optimize rubber yields as well as the ease of harvesting and processing. Marker-assisted selection is a powerful method to improve precision and efficiency during plant breeding. Numerous quantitative trait loci (QTLs) and/or associated genes for a variety of plant species and traits have been identified to assist breeders in plant selection as early as the seedling stage, for example in rice (Yonemaru et al., 2010). Suitable markers have not yet been identified in *T. koksagyhz*. Most studies thus far have focused on dandelion metabolism (Schmidt et al., 2010; Epping et al., 2015; Pütter et al., 2017; Stolze et al., 2017; Niephaus et al., 2019) whereas few have considered growth characteristics (Munt et al., 2012; Wieghaus et al., 2019) or field trials (Arias et al., 2016a; Kreuzberger et al., 2016; Eggert et al., 2018; Keener et al., 2018). Most of the relevant studies have aimed to improve yields or gain knowledge about agronomic performance.

To facilitate the breeding of new dandelion varieties with improved traits, we developed a MACE-based transcriptomic approach to identify candidate genes involved in root growth. Three sets of plants with diverse root morphotypes were harvested and the roots were divided into upper (proximal) and root tip (distal) segments. The primary root volume and root length differed significantly between the morphotypes, whereas the rubber content was consistent. By pooling the plants, we increased the likelihood of identifying specific transcripts associated with root morphology in a heterogenous set of wild-type plants while minimizing differences in the genetic background. This reduced the number of DEGs we recovered, but ensured that they were associated with a given root phenotype. The analysis of differential gene expression in the resulting datasets produced four sets of candidate genes influencing primary root volume or length in the upper root or root tip.

Our transcriptomic pipeline allowed the identification of multiple genes for specific root traits. Several of these were assigned to transcription factors, hormone pathway/responsive factors and cell wall/glucan biosynthesis/degradation proteins. We found that genes in these groups, encoding products such as Protein EXORDIUM and cellulose synthase A catalytic subunit 6 (UDP-forming) are already known to play a role during root development, but also highlighted several interesting genes which are yet to be evaluated for a putative role in root growth and morphogenesis.

We selected three candidate genes for verification in single *T. koksagyhz* plants from a new set of wild-type plants grown under simulated field conditions. Because of the heterogeneity of the plants used, we did not expect the correlation to be very high. Many factors determine the root phenotype and our candidate genes might contribute to it. Therefore, we think that a weak correlation might still be reasonable when combined with other identified DEGs. Nevertheless, two of the candidate genes, encoding proteins resembling β-1,3-glucanase 11 (TkBG11) and jasmonate ZIM-domain protein 1 (TkTIFY10A/JAZ1), were confirmed by qRT-PCR as more strongly expressed in thinner and longer roots, respectively. In contrast, there was no correlation in the expression of TkAOS3, encoding a putative allene oxide synthase.

The TkBG11 protein may affect the architecture of plasmodesmata during root development, influencing the way all plant cells connect to their neighbors. The permeability of these membrane-lined microscopic channels is controlled by interactions between callose synthases and β-1,3-glucanases, which regulate the accumulation of callose in the surrounding cell walls and thus determine the aperture size (Wolf et al., 1991; Levy et al., 2007; Vatén et al., 2011; De Storme and Geelen, 2014). Plasmodesmata play an important role in cell–cell communication via the symplastic transport of auxin (Liu et al., 2017; Mellor et al., 2020), transcription factors such as SHORT ROOT (Nakajima et al., 2001; Kim et al., 2002, 2005; Carlsbecker et al., 2010; Vatén et al., 2011), LEAFY (Wu et al., 2003), KNOTTED1 (Lucas et al., 1995) and PLETHORA 2 (Mähönen et al., 2014), and microRNAs such as miRNA165/6 (Carlsbecker et al., 2010; Vatén et al., 2011). For example, the quiescent center in the meristematic zone of the root tip maintains a single layer of immediately adjacent stem cells by signaling through the plasmodesmata (Barclay et al., 1982; Van Den Berg et al., 1997; Stahl et al., 2013; Liu et al., 2017). Likewise, lateral root development in *A. thaliana* depends on symplastic connectivity (Benitez-Alfonso et al., 2013). Several pathogen-related β-1,3-glucanases of the PR-2 family not only play a role in defense by degrading fungal cell walls (Thanseem et al., 2005; Liu et al., 2009) but also by inhibiting the spread of viruses through plasmodesmata (Zavaliev et al., 2013). Following the hypothesis that TkBG11 plays a role in callose homeostasis in root cell plasmodesmata, its impact on root development may reflect the depletion of callose in the plasmodesmata to increase the trafficking of hormones and/or miRNA.

TkTIFY10A/JAZ1 is likely to play a role during root elongation because it was strongly expressed in long roots and this was confirmed by qRT-PCR in plants grown under field conditions. In *A. thaliana*, the knockdown of JAZ1 sensitized the plants to methyl jasmonate (MeJA), which inhibited primary root growth but increased the number of lateral roots (Grunewald et al., 2009). In contrast, the overexpression of JAZ1 or knockout of COI1 increased the hypocotyl length under far-red light (Robson et al., 2010; Liu and Wang, 2020). Furthermore, JAZ1 is upregulated by auxin (Grunewald et al., 2009). These findings indicate a similar role for JAZ1 during root development in *T. koksaghyz*, because stronger expression was observed in plants with significantly longer roots. However, it is unclear whether a jasmonate-mediated response to pathogens resulted in shorter roots in pool B.

Although qRT-PCR did not confirm the correlation between primary root length and the expression of TkAOS3 as suggested by the transcriptomic data, the gene may still influence root development in the context of defense against pathogens, and this may explain the discrepancy between our two sets of plants (one grown in the greenhouse and the other exposed to pathogens in the environment). This hypothesis is also supported by its high expression in latex. The products of the 9-LOX pathway have been proposed to mediate defense responses in plants (Vellosillo et al., 2007; Gao et al., 2008; León Morcillo et al., 2012; Vicente et al., 2012; Marcos et al., 2015; Christensen et al., 2016; Morcillo et al., 2016). The strong expression of TkAOS3 therefore hints at a thin-root phenotype resulting from interactions with pathogens during root development. Interestingly, *LeAOS3* is not detected in the roots of *jai1/myc2* tomato mutants (Itoh et al., 2002). MYC2 regulates several early jasmonate response genes as a component of plant defenses (Yadav et al., 2005; Grunewald et al., 2009; Kazan and Manners, 2013), which indicates a link between the 9-LOX pathway and jasmonate defense system (Itoh et al., 2002). Given that our TIFY10A/JAZ1 homolog, which is more strongly expressed in long roots, may repress MYC2 (Chini et al., 2007; Fernández-Calvo et al., 2011) and that our AOS3 homolog is more strongly expressed in thin roots, the expression profile is at least confirmed for pools A and B. But because pool C is characterized by the stronger expression of both the AOS3 and TIFY10A/JAZ1 homologs, there appears to be a more complex relationship between these pathways.

## Conclusion

We have provided initial insight into the expression of genes in the upper root and root tip of *T. koksaghyz* plants by identifying genes that are differentially expressed between diverse root phenotypes. Several candidate genes were identified and the expression of three of them was tested by qRT-PCR in a new set of wild-type plants grown under field conditions, correlating with the transcriptomic data in two cases and confirming the validity of our method. We also generated a set of candidate genes suitable for further research and subsequent use in marker-assisted breeding. The results strengthen our strategy of first identifying bulk DEGs by comparing pools of plants with similar phenotypes, followed by a verification on a separate set of plants. The latter step reduces the overall number of candidate genes but homes in on the most promising candidates. In this case, the candidate genes TkBG11 and TkTIFY10A may be useful for the optimization of root phenotypes in *T. koksaghyz*, which entails roots of medium length with a high biomass preferably in the upper root part. Varieties showing this root phenotype are likely to be more suitable for harvesting and processing, thereby optimizing not only the yield of rubber and inulin but also the recovery of these valuable products.

## Data Availability

The datasets presented in this study can be found in online repositories. The names of the repository/repositories and accession number(s) can be found below: NCBI SRA BioProject, accession no: PRJNA783530.
