# Supplementary figures and images for: Comparative Transcriptome Analysis in Taraxacum koksaghyz to Identify Genes that Determine Root Volume and Root Length
Source: Front Genet. 2022 Jan 24;12:784883. doi: 10.3389/fgene.2021.784883 (PMC8819189; doi:10.3389/fgene.2021.784883)

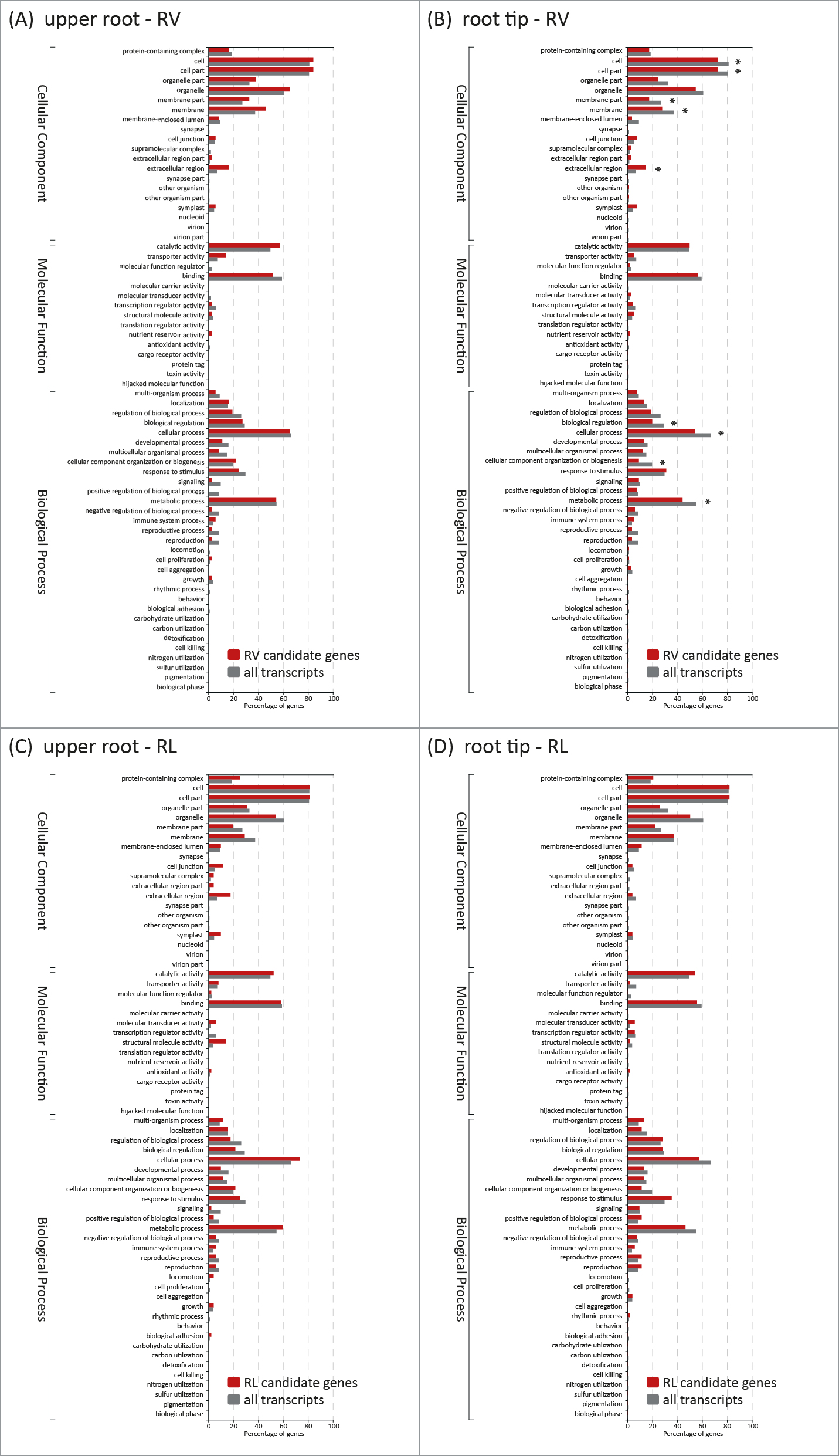

Supplement: Supplementary file 2 [file Image3.JPEG]

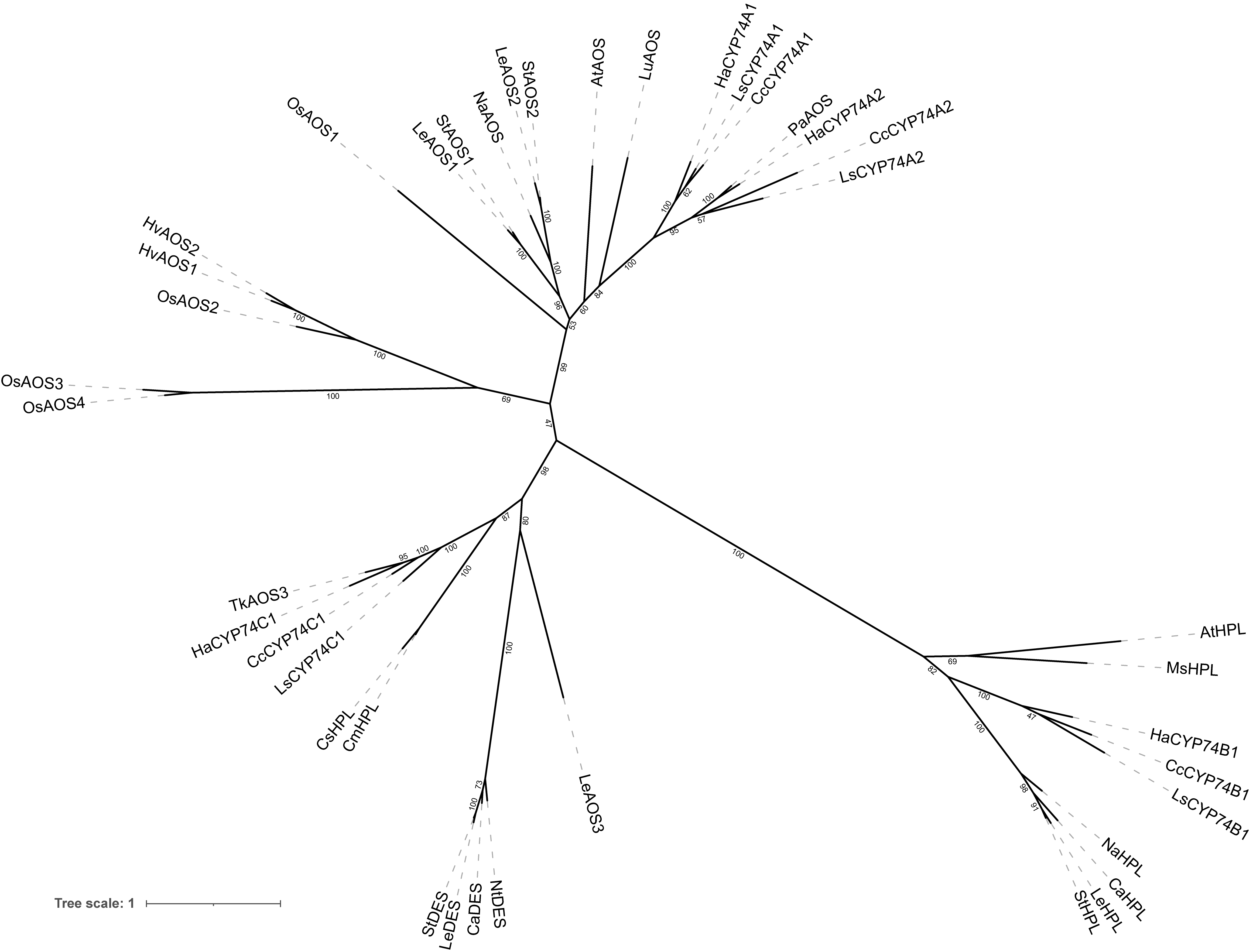

Supplement: Supplementary file 4 [file Image6.TIF]

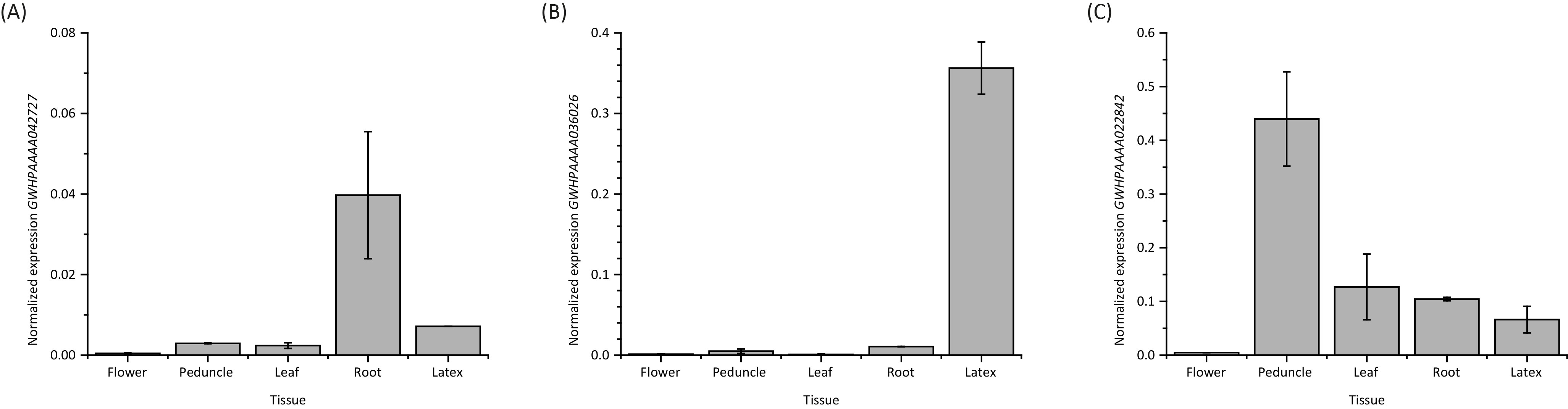

Supplement: Supplementary file 5 [file Image4.JPEG]

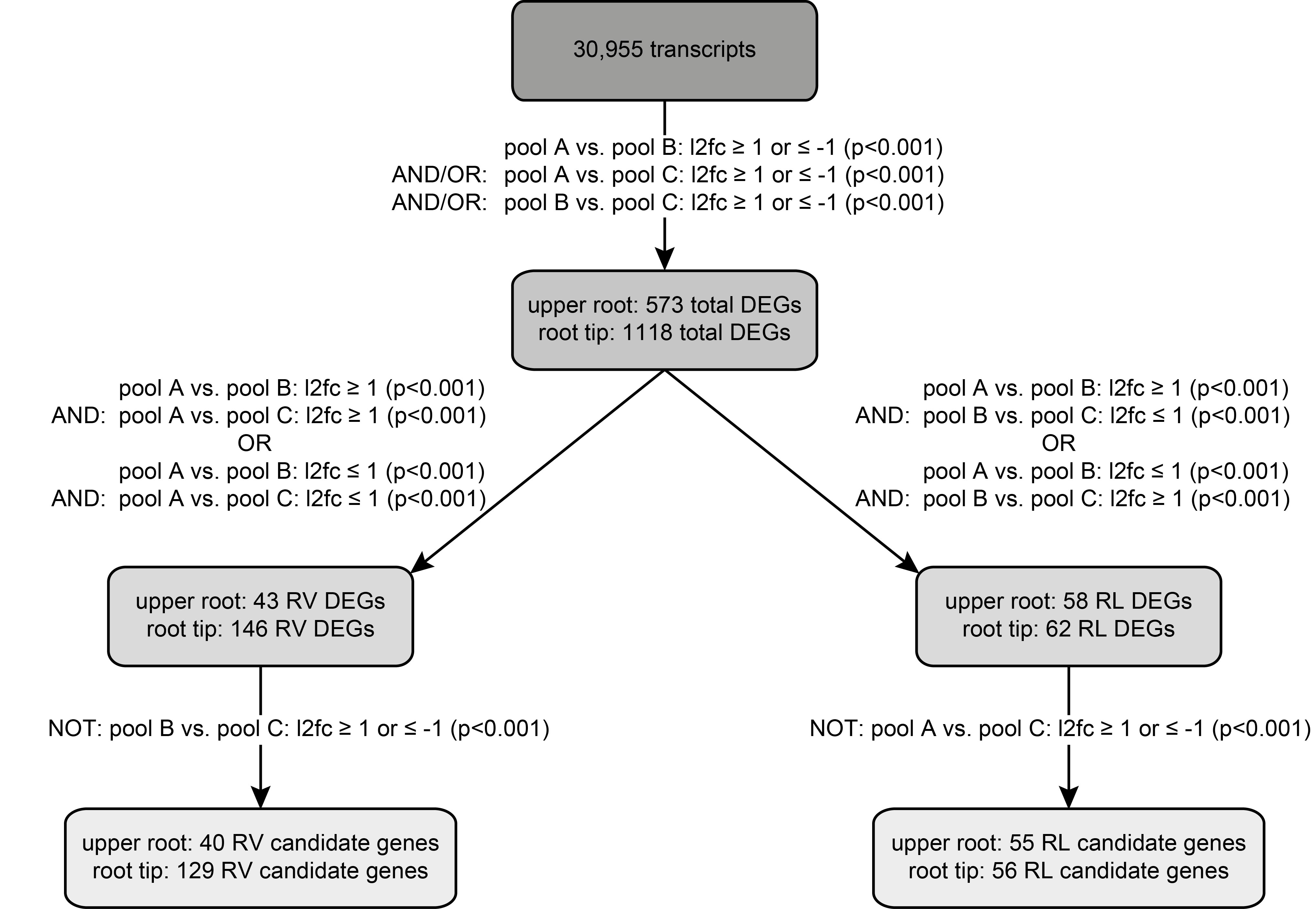

Supplement: Supplementary file 6 [file Image2.JPEG]

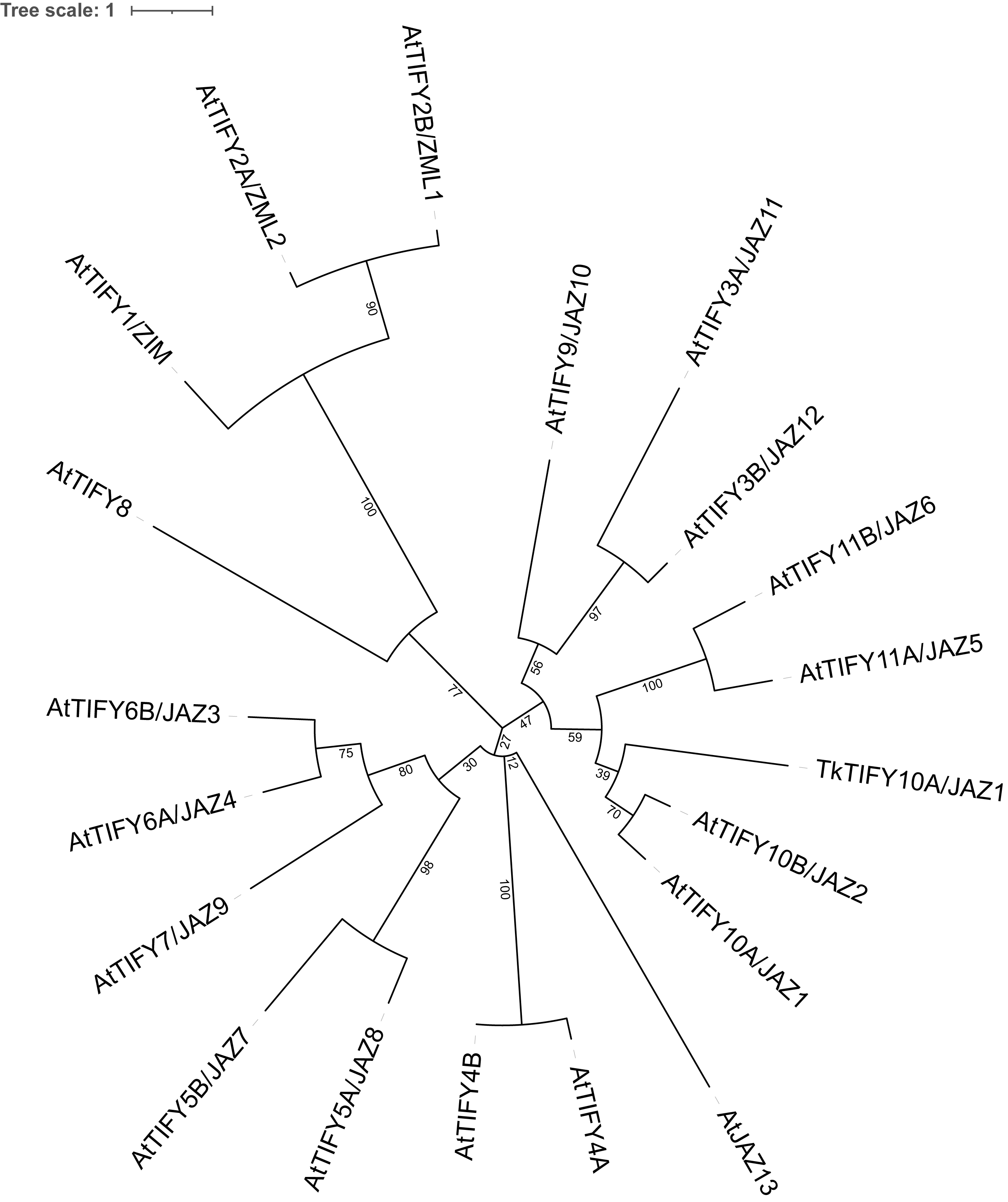

Supplement: Supplementary file 7 [file Image7.TIF]

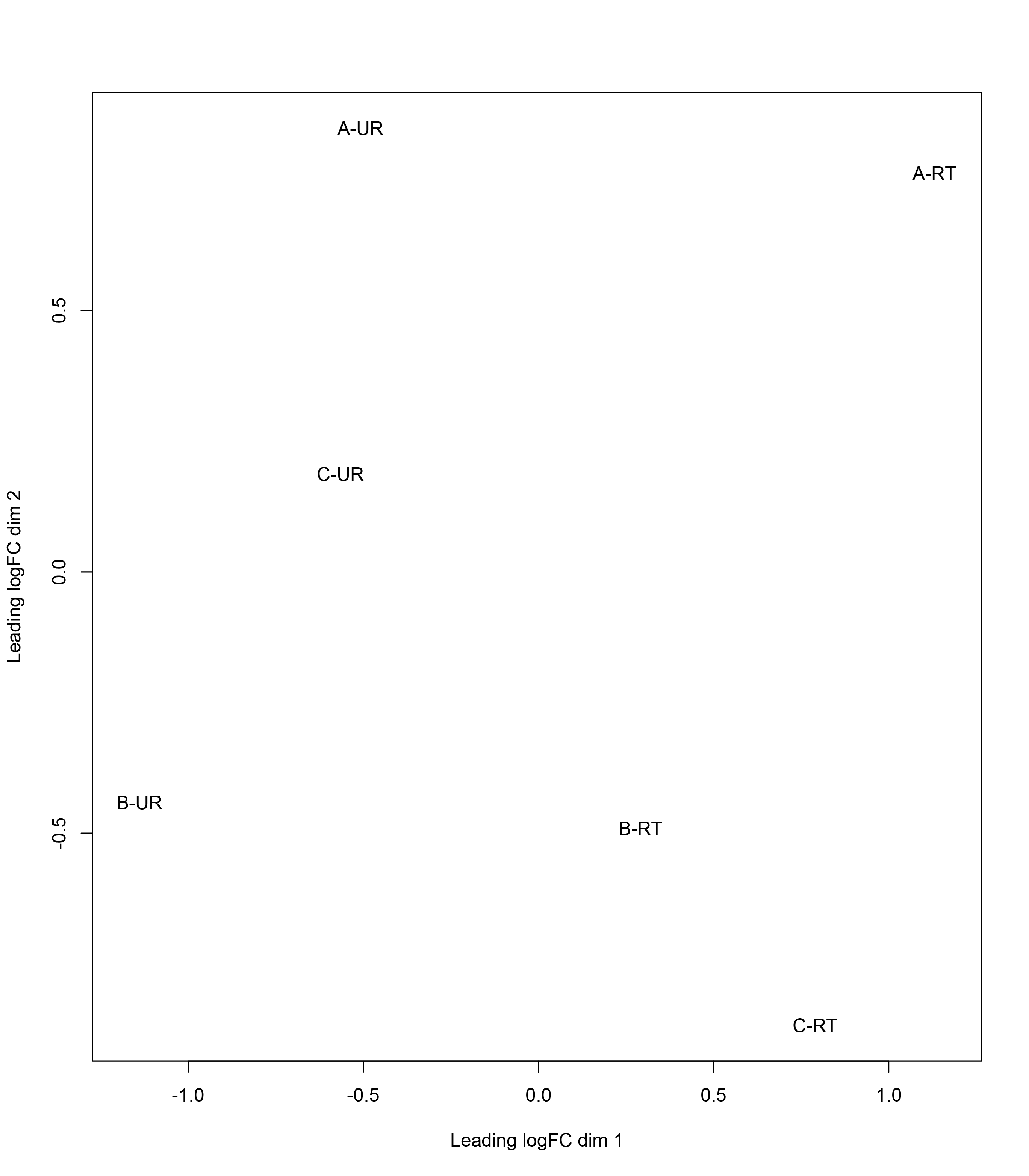

Supplement: Supplementary file 11 [file Image1.PNG]

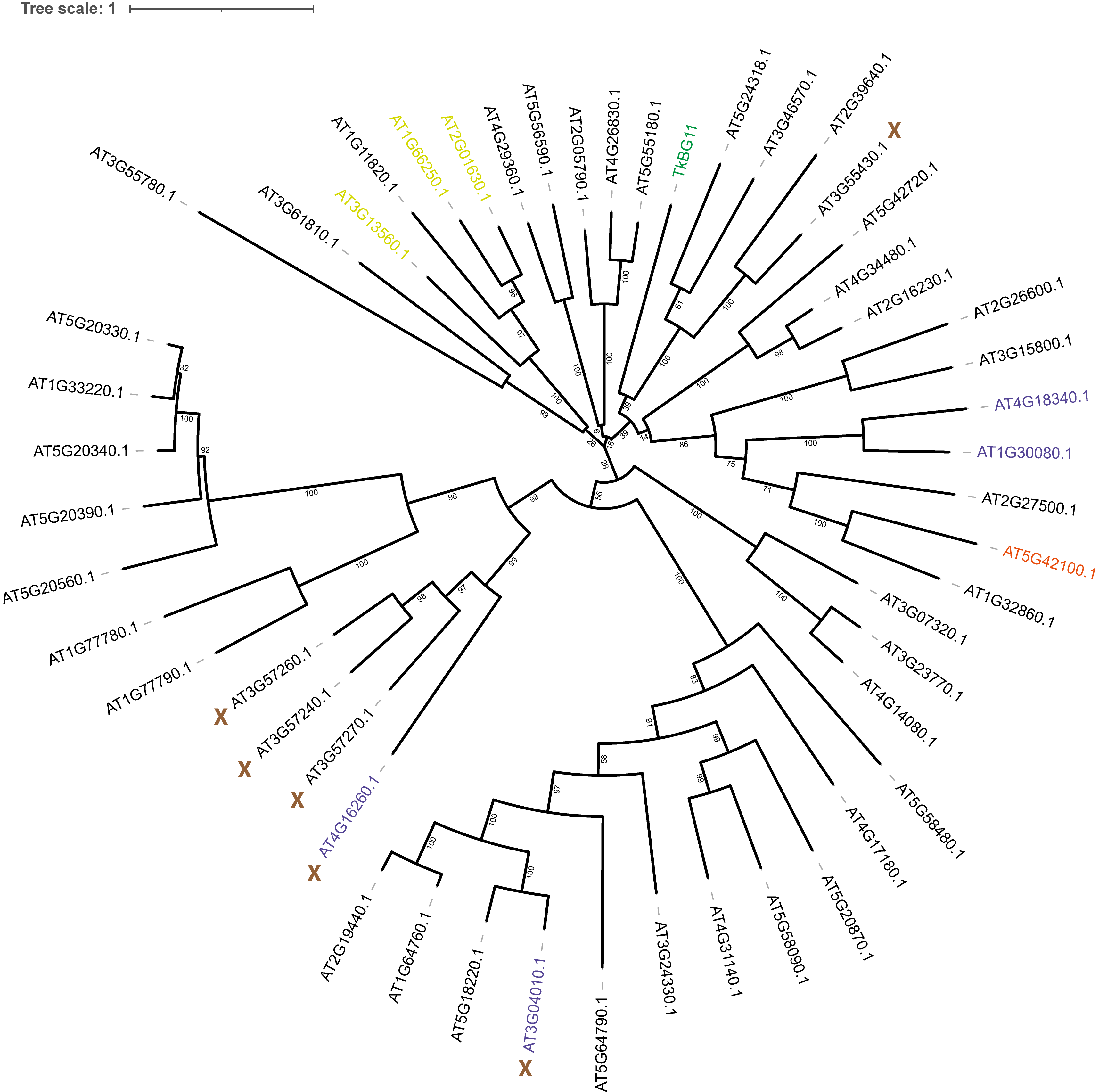

Supplement: Supplementary file 12 [file Image5.TIF]
